# Evaluation of polycyclic aromatic hydrocarbon (PAH) mass concentrations in smoke generated during pork belly grilling over charcoal

**DOI:** 10.1016/j.fochx.2026.103558

**Published:** 2026-01-19

**Authors:** Yong-Hyun Kim, Sung-Hwan Kim

**Affiliations:** a Department of Environment & Energy, Jeonbuk National University, 567 Baekje-daero, Deokjin-gu, Jeonju-si, Jeonbuk State 54896, Republic of Korea; b Department of Environmental Engineering, Jeonbuk National University, 567 Baekje-daero, Deokjin-gu, Jeonju-si, Jeonbuk State 54896, Republic of Korea; c Soil Environment Research Center, Jeonbuk National University, 567 Baekje-daero, Deokjin-gu, Jeonju-si, Jeonbuk State 54896, Republic of Korea; d Jeonbuk Branch Institute, Korea Institute of Toxicology, Jeongeup-si, Jeonbuk State 56212, Republic of Korea

**Keywords:** Polycyclic aromatic hydrocarbons (PAHs), Benzo[*a*]pyrene (BaP), Pork belly, Charcoal, Thermal desorption

## Abstract

Charcoal grilling emits substantial polycyclic aromatic hydrocarbons (PAHs), yet exposure assessment is complicated by challenging short-duration smoke sampling. We quantified PAHs using thermal desorption-gas chromatography-mass spectrometry, achieving a benzo[*a*]pyrene (BaP) detection limit of 5 pg Sm^−3^ from a 360 L air sample. Grilling pork belly over charcoal produced a total EPA PAH concentration of 98.4 μg Sm^−3^, with BaP alone reaching 1431 ng Sm^−3^—contrasting sharply with 0.26 μg Sm^−3^ in ambient air. Combustion without meat yielded BaP concentrations from 0.67 ng Sm^−3^ (hardwood) to 953 ng Sm^−3^ (chaff), highlighting the decisive influence of fuel type. Compared to the WHO/EU guideline (1 ng Sm^−3^), these data demonstrate that charcoal grilling generates extreme inhalation risks, exceeding permissible limits by over 1400-fold. The results underscore the urgent need for emission control and safer grilling standards.

## Highlights

• PAH emissions were evaluated during charcoal combustion and pork belly grilling.
• TD-GC-MS detected benzo[*a*]pyrene at 4.65 pg Sm^−3^ in 360 L air samples.
• Benzo[*a*]pyrene ranged from 0.67 to 953 ng Sm^−3^ by charcoal type and origin.
• Grilling pork belly over charcoal yielded 98.4 μg Sm^−3^ of 16 EPA-regulated PAHs.
• PAH emissions varied with charcoal type and increased during grilling.

## Graphical abstract

**Figure:**
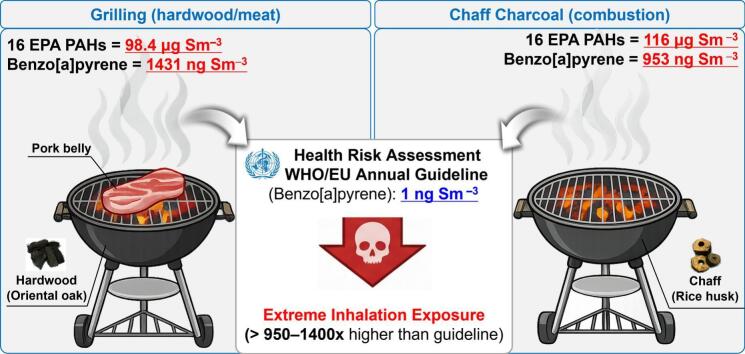

## 1 Introduction

Polycyclic aromatic hydrocarbons (PAHs) in the ambient air have been identified as potential causes of adverse health effects in humans. Dermatological contact with PAHs elicits allergic reactions and dermatitis that can manifest as skin irritation and inflammation (Hendricks et al., 2020; Ju & Zouboulis, 2016; Yanagisawa et al., 2021). Inhalation exposure to PAHs has been linked to an increased risk of developing respiratory conditions, such as bronchitis and asthma (Kim et al., 2013; Låg et al., 2020; Ravanbakhsh et al., 2023), and a markedly elevated risk of developing cancers, particularly lung cancer (Kim et al., 2013; Sarigiannis et al., 2015; Stading et al., 2021). PAHs absorbed into the human body can disrupt immune system functioning, reducing the ability of the body to combat infections and diseases (Hew et al., 2015; Yao et al., 2019). Exposure to PAHs in pregnant women has been associated with developmental abnormalities in the fetus, with impaired growth and long-term health complications observed (Dai et al., 2023; Tartaglione et al., 2023; Yang et al., 2020). The wide-ranging and severe health impacts of PAHs have led to their designation as hazardous air pollutants, and governments globally have placed strict regulatory controls on these pollutants to minimize public exposure.

Numerous countries have implemented strategies to mitigate atmospheric PAH concentrations by establishing regulatory emission limits. For example, the World Health Organization (WHO) and European Union (EU) set an annual average concentration limit of 1 ng m^−3^ for benzo[*a*]pyrene (BaP) in ambient air (Cavanagh et al., 2012; Jasiura et al., 2023; Porwisiak et al., 2023), and some nations have pursued stricter measures, aiming to maintain PAH concentrations below detectable limits, which is often constrained by the sensitivity of the current analytical instruments (Boehm, 1964; Kalf et al., 1997; Wise et al., 2015). Despite these regulations, PAHs are persistently emitted into the atmosphere from industrial processes, vehicular emissions, and waste incineration (Cui et al., 2022; Dat & Chang, 2017; Han et al., 2019; Lin et al., 2022). Recently, cooking has emerged as a significant yet often overlooked source of human PAH exposure (Deng et al., 2023; Kuye & Kumar, 2023; Lin et al., 2022). Unlike other sources, cooking exposes individuals to potentially elevated levels of PAHs in proximity. The variability in the PAH types and concentrations emitted during cooking depends heavily on the ingredients and cooking methods used, complicating efforts to establish comprehensive regulations. Addressing this challenge requires focused research to identify and quantify the PAHs released during cooking as a critical first step toward developing emission control strategies. As PAHs are predominantly produced via the incomplete combustion of hydrocarbons, grilling warrants particular attention due to its high potential for PAH generation. Consequently, prioritizing the evaluation of PAH emissions from grilling and similar cooking practices is essential to understanding and mitigating the risk of exposure.

Grilling meat is a widespread culinary practice globally; however, it is a significant source of PAHs released into the atmosphere (Arar et al., 2022; Badyda et al., 2022; Duedahl-Olesen & Ionas, 2022). PAHs are formed when the fat in meat decomposes at high temperatures, releasing hazardous compounds (Das et al., 2023; Olatunji et al., 2014). The use of charcoal as a grilling fuel exacerbates PAH emissions due to incomplete fuel combustion (Arar et al., 2022). Furthermore, fat that drips onto the burning charcoal combusts, producing additional PAHs, particularly in charcoal-cooked meat preparations (Lee et al., 2016; Viegas et al., 2014). These emissions underscore the need for a comprehensive evaluation of human exposure to PAHs during grilling.

The accurate measurement of PAH mass concentrations during grilling is essential for a precise assessment of the associated health risks. However, systematic investigations into the airborne PAHs generated during grilling are limited. Most previous studies have focused on the relative PAH content in grilled meat (Kalteh et al., 2024) and the health risks associated with its consumption (Adeyeye, 2020; Lu et al., 2024). Research addressing the inhalation exposure to airborne PAHs emitted during grilling is scarce, representing a significant knowledge gap.

The paucity of data on airborne PAHs during grilling is partly due to the technical challenges involved in PAH detection. PAHs in ambient air typically exist at sub-ng m^−3^ levels, which is far lower than those of common air pollutants such as benzene, toluene, SO_2_, and NO_2_. The detection of trace-level PAHs thus requires large volumes of air (Kim & Kim, 2015). Conventional methods often employ high-volume air samplers (flow rates of 0.2–1.5 m^3^ min^−1^); however, the sampling must continue for 24 h or longer to collect at least 200 m^3^ of air, ensuring detectable PAH concentrations (Kalisa et al., 2022). As grilling is typically performed for only a few hours, such extended sampling is impractical. Additionally, although PAH concentrations may be significantly higher than ambient levels near grilling areas, levels still need to be quantitatively distinguished from background concentrations. Simple reductions in the sampling time are insufficient to overcome these challenges. Therefore, it is imperative to develop optimized sampling, pretreatment, and analytical methods tailored to the short-term high-intensity PAH emissions produced during grilling.

To analyze trace-level PAHs in ambient air, this study employed a previously developed solid-phase adsorption-based method for sampling and pretreatment (Kim & Kim, 2015). The utilized system consisted of two sequentially connected sorbent tubes, one of which was packed with quartz wool and the other with Carbopack C, and a mini-vacuum pump was attached downstream of the Carbopack C tube to draw air through the system. This configuration enabled the selective collection of particulate PAHs in the quartz wool tube and gas-phase PAHs in the Carbopack C tube. The captured PAHs were then analyzed using optimized thermal desorber-gas chromatography-mass spectrometry (TD-GC-MS), facilitating the thermal desorption, separation, identification, and quantification of the compounds. This analytical approach is particularly effective for quantifying low-concentration PAHs because it minimizes loss during pretreatment. The detection limit of the system is less than 30 pg by mass, enabling the quantification of PAHs at concentrations as low as 0.3 ng m^−3^ with a sampling volume of only 100 L. This method allowed the successful collection and quantification of the trace PAHs emitted during short-term grilling events lasting less than one hour.

The sorbent tube-based TD-GC-MS system enabled accurate sampling, pretreatment, and analysis of the air samples, allowing the mass concentrations of PAHs released during grilling with charcoal and gas burners to be assessed together with the mass concentrations of PAHs emitted solely from fuel combustion. The results were used to elucidate the relative contributions of the grilling process and fuel type to the total PAH emissions. The mass-based quantification of the PAHs generated during grilling underscores the inhalation exposure risks associated with such activities and provides valuable insights for developing strategies to mitigate health hazards.

## 2 Materials and methods

### 2.1 Target PAHs

The study focused on the analysis of 16 priority PAHs: naphthalene (NAP), acenaphthylene (ACY), acenaphthene (ACE), fluorene (FLU), phenanthrene (PHEN), anthracene (ANTH), fluoranthene (FLTH), pyrene (PYR), benzo[*a*]anthracene (BaA), chrysene (Chrys), benzo[*b*]fluoranthene (BbF), benzo[*k*]fluoranthene (BkF), BaP, indeno[1,2,3-*cd*]pyrene (IP), dibenzo[*a*,*h*]anthracene (DbahA), and benzo[*g*,*h*,*i*]perylene (BghiP). These compounds have been identified by the US EPA due to their carcinogenicity, toxicity, environmental persistence, and potential for bioaccumulation (Table S1).

### 2.2 Experimental approaches

**Fig. 1:**
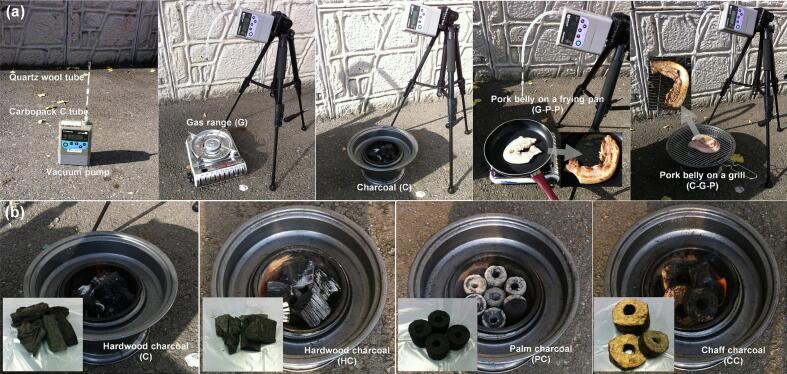
Photographs showing the method used for smoke and air sampling. (a) Different grilling methods (Exp 1) and (b) Different charcoal types (Exp 2).

The mass concentrations of PAHs generated during grilling were investigated using two distinct experimental setups (Exps 1 and 2) (Fig. 1 and Table 1). Meat samples were obtained from a local butcher shop in Seoul, Republic of Korea. In Exp 1, PAHs emitted during grilling on charcoal with a heated pan were captured using sorbent tubes and analyzed with a TD-GC-MS system. The mass concentrations of PAHs emitted during grilling were then compared with those detected in ambient air to evaluate how grilling affected PAH emissions. Exp 2 focused on the PAHs emitted solely from the combustion of different types of charcoal, allowing the influence of charcoal type on atmospheric PAH emissions to be assessed by analyzing the mass concentrations of PAHs released during the burning process.

**Table 1:** Key experimental conditions used in the evaluation of PAH mass concentrations based on different meat grilling methods: experimental materials and PAH sampling conditions.

Order	Sample code^a^	Material					Sampling					
		Target air sample	Pork belly weight (g)	Charcoal material	Charcoal weight (g)		Sampling method^b^	Absorbent^c^	Sampling flow rate (L min^−1^)	Sampling time (min)	Sampling volume (L)	Temperature (°C)
A. Exp 1: Assessment of PAH concentrations arising from charcoal-grilled meat (pork belly)												
1	Amb	Ambient air	-^a^	-	-		Sorbent tube sampling	Quartz wool and Carbopack C	3	120	360	15 ± 2.2
2	G	Smoke generated by the butane gas flame	-	-	-					30	90	19.1 ± 1.8
3	C	Smoke generated by a charcoal flame	-	Hardwood (Oriental oak)	101 ± 2.05					30	90	29.2 ± 4.1
4	G-P-P	Smoke produced from cooking pork belly in a frying pan over a gas flame	201 ± 2.31	-	-					10	30	18.8 ± 3.5
5	C-G-P	Smoke produced from grilling pork belly on a charcoal grill	200 ± 1.21	Hardwood (Oriental oak)	99.5 ± 1.55					10	30	28.5 ± 3.3
B. Exp 2: Evaluation of PAH concentrations emitted during charcoal combustion												
6	C	Smoke generated by a charcoal flame	-	Hardwood (Oriental oak)	101 ± 2.05		Sorbent tube sampling	Quartz wool and Carbopack C	3	30	90	29.2 ± 4.1
7	HC		-	Hardwood (Oriental oak)	98.2 ± 3.12				3	2 and 20	5 and 50	26.7 ± 2.64
8	PC		-	Palm (Palm tree)	100 ± 2.55					2 and 20	5 and 50	29.8 ± 1.91
9	CC		-	Chaff (Rice husk)	99.5 ± 2.11					1 and 10	2.5 and 25	26.7 ± 1.61

^a^Not available

^b^Ambient air (Amb); Gas range (G); Charcoal (C; Gangwon-do, Republic of Korea); Gas-Pan-Pork belly (G-P-P); Charcoal-Grill-Pork belly (C-G-P); Hardwood Charcoal (HC; Chungcheongbuk-do, Republic of Korea); Palm Charcoal (PC; Vietnam), and Chaff Charcoal (CC; Vietnam).

^c^The atmospheric specimens were processed through a sorbent tube that was designed to enhance the adsorptive entrapment of PAHs from the studied samples.

^d^1st tube: Quartz wool 25 mg; 2nd tube: Quartz wool 10 mg + Carbopack C (60/80) 50 mg; Sampling tube holder material: Quartz (length: 90 mm, OD: 6.4 mm, and ID: 4.2 mm).

#### 2.2.1 Generation and collection of cooking smoke based on different grilling methods (Exp 1)

Exp 1 was performed to elucidate the mass concentrations of PAHs emitted from butane gas and charcoal flames together with the PAHs generated by grilling pork belly (Fig. 1 and Table 1). Ambient air PAH concentrations were measured to establish background levels for comparison. Five experimental setups were employed, with each performed in triplicate: Amb (ambient air), G (smoke from butane gas flame), C (smoke from charcoal flame), G-P-P (smoke from cooking pork belly in a frying pan over a gas flame), and C-G-P (smoke from grilling pork belly on a charcoal grill).

Samples were collected using a series of sorbent tubes; the first tube was packed with quartz wool and the second with Carbopack C (mesh size 60/80, Supelco, USA). The front end of the sorbent tube was positioned near the sample source, while the rear end was connected to a mini vacuum pump equipped with a mass flow controller (MP-Σ300, Sibata, Japan). Air samples were then drawn through the sorbent tubes to allow the adsorption of PAHs onto the sorbent materials. The flow rate was maintained consistently at 3 L min^−1^ in all experiments, with sampling durations adjusted for each sample type.

For the Amb sample, ambient air was drawn through the sorbent tube at 3 L min^−1^ over 120 min, resulting in a total sampling volume of 360 L. For the G sample, smoke was generated by combusting butane gas with a gas burner, while for the C sample, smoke was produced by igniting 101 ± 2.05 g of hardwood charcoal (Oriental oak, Gangwon-Do, Republic of Korea). Smoke from both the G and C samples was collected for 30 min at 3 L min^−1^, yielding a total sampling volume of 90 L per sample. The setups for G and C were otherwise similar to those for G-P-P and C-G-P, but the latter involved cooking pork belly. A frying pan was placed over a gas flame for G-P-P, and a grill was placed over a charcoal flame for C-G-P. Approximately 200 g of pork belly was cooked, generating smoke that was collected over 10 min at 3 L min^−1^. Total sampling volumes of 30 L were obtained for both G-P-P and C-G-P—.

#### 2.2.2 Generation and collection of charcoal smoke based on different charcoal types (Exp 2)

Exp 2 was conducted to analyze and compare the mass concentrations of PAHs in the smoke generated from four distinct types of charcoal (Fig. 1 and Table 1). The PAH concentrations for ambient air (background sample) and charcoal (C sample) were derived from the data collected in Exp 1. All experiments, including smoke generation and PAH analyses for the various charcoal types, were performed in triplicate. Sample codes indicate C (hardwood, oriental oak, Gangwon-Do, Republic of Korea), HC (hardwood, oriental oak, Chuncheongbuk-Do, Republic of Korea), PC (Palm, Palm tree, Vietnam), and CC (chaff, rice husk, Vietnam).

For each sample, approximately 100 g of charcoal was ignited, and the resulting smoke was collected using the two-stage sorbent tube system, with both particulate- and gas-phase PAHs obtained. Smoke samples were drawn through the sorbent tubes at a constant flow rate of 3 L min^−1^, and the sampling durations were adjusted according to the type of charcoal to achieve specific sampling volumes.

For the HC and PC samples, sampling durations of 2 and 20 min were used, corresponding to total sampling volumes of 5 and 50 L, respectively, while for the CC samples, durations of 1 min and 10 min were applied, resulting in total sampling volumes of 2.5 L and 25 L, respectively. These two distinct sampling volumes were employed to ensure that the PAH masses detected in the HC, PC, and CC samples remained within the calibration range of the analytical system, enabling precise and reliable concentration calculations.

### 2.3 Instrumental system

The PAHs adsorbed on the sorbent tubes were thermally desorbed using a TD (TD-20, Shimadzu, Japan) and transferred to a GC (GC-2010, Shimadzu, Japan) coupled with an MS (GCMS-QP2010 ultra, Shimadzu, Japan) for separation and quantification.

To facilitate the desorption of PAHs, sorbent tubes were heated to 290 °C for 7 min with helium gas (purity >99.999%) flowing at 100 mL min^−1^. The desorbed PAHs were then readsorbed onto a cold trap within the TD containing 10 mg of quartz wool and 50 mg of Tenax TA. The cold trap was then heated to 300 °C for 5 min, and helium gas flowing at 16 mL min^−1^ was used to transfer the PAHs into the gas chromatograph. PAHs were then separated using a DB-5MS capillary column (DB-5MS, Agilent J&W, USA) in a GC oven that was initially set at 80 °C for 5 min, then increased at 20 °C min^−1^ to a final temperature of 300 °C, and finally maintained at 300 °C for 24 min, with a total analysis time of 40 min. The separated PAHs were ionized using a mass spectrometer via electron ionization at 70 eV, and quantification was performed using total ion chromatograms (35–600 *m*/*z*), with the extracted ion chromatograms utilized for more precise quantification (Kim & Kim, 2015). The ion source and interface temperatures of the MS were both set at 280 °C (Table S2).

### 2.4 Calibration and quality control (QC)

#### 2.4.1 Preparation of PAH working standards

Calibration and QC data for the 16 target PAHs were generated using working standards prepared by serial dilution of a primary standard (PS) solution (EPA 610 Polynuclear Aromatic Hydrocarbon Mixture, Supelco, USA). The PS contained 9.94 ± 0.08 ng μL^−1^ of each PAH in methanol. Seven working standards were prepared in 2 mL amber vials by mixing 20–2000 μL of the PS with methanol (0–1980 μL) to achieve final concentrations ranging from 0.099 ± 0.001 ng μL^−1^ to 9.94 ± 0.08 ng μL^−1^ (Table S3).

#### 2.4.2 Analysis of PAH working standards

Working standards were introduced directly into the sorbent tubes to simulate vaporization and adsorption distribution, followed by analysis using the TD-GC-MS system. Two types of sorbent tubes were employed: one packed with 25 mg of quartz wool and the other with 50 mg of Carbopack C. For consistency with the ambient air and smoke sample collection, additional calibration and QC data were obtained using sorbent tubes packed with both quartz wool and Carbopack C.

Teflon tubing was used to connect sorbent tubes to the inlet, and a filter tube containing Carbopack C was connected via silicone tubing to the outlet of a nitrogen gas cylinder (purity >99.999%). A liquid syringe was used to inject 1 μL of each working standard into the sorbent tube inlet, with additional volumes of 2 μL and 5 μL also injected for the 7th working standard. Nitrogen gas flowing at 3 L min^−1^ for 3 min was utilized to facilitate the vaporization of PAHs and their adsorption onto the sorbent material during injection. The sorbent tubes containing the adsorbed PAHs were then analyzed using the TD-GC-MS system following the procedure detailed in Section 2.3.

## 3 Results and discussion

### 3.1 Calibration and QC results for analytical system

**Fig. 2:**
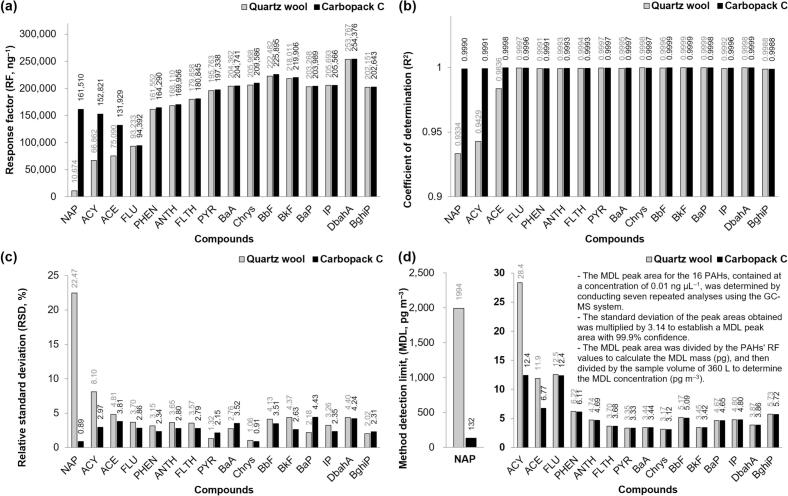
Calibration and QC results for PAH sampling and analytical system. (a) Response factor (RF, ng^−1^), (b) Coefficient of determination (R^2^), (c) Relative standard deviation (RSD, %), and (d) Method detection limit (MDL, pg m^−3^).

Comprehensive calibration and QC data were obtained to quantify the 16 PAHs emitted during grilling accurately. Key metrics included: (1) the response factor (RF, ng^−1^), (2) the coefficient of determination (R^2^), (3) analytical repeatability (relative standard deviation, RSD, %), and (4) the method detection limit (MDL, pg m^−3^) (Fig. 2).

The results showed RF values that were 1.73–15.1 times higher in the Carbopack C tubes than in the quartz wool tubes for NAP, ACY, and ACE; however, the RF values were consistent between the two sorbent tube types for the remaining 13 PAHs, with RSDs of less than 2%. The lower RF values for NAP, ACY, and ACE in the quartz wool tubes were attributed to their lighter molecular weights (with two or fewer aromatic rings), which resulted in reduced capture efficiency for these particulate-phase PAHs. The RF values for the remaining 13 PAHs ranged from 93,233 (FLU, quartz wool) to 254,376 (DbahA, Carbopack C) ng^−1^.

Calibration curves obtained using Carbopack C tubes exhibited excellent linearity, with R^2^ values exceeding 0.99 for all PAHs. In contrast, the quartz wool tubes showed lower linearity only for NAP, ACY, and ACE, with R^2^ values of 0.9334, 0.9429, and 0.9836, respectively. These results again indicate reduced capture efficiency and reliability for the capture of lighter PAHs by the quartz wool tubes, undermining the consistency of their detection across various mass concentrations.

The repeatability of the PAH analysis, reflected by the RSD values, was closely aligned with the trends observed for the R^2^ values. The Carbopack C tubes demonstrated exceptional repeatability for all PAHs, with RSD values below 5%; however, higher variability for NAP, ACY, and ACE was observed in the quartz wool tubes, with RSD values of 22.47, 8.10, and 4.81%, respectively. In particular, the repeatability of NAP was notably poor when analyzed using the quartz wool tubes.

The MDL for PAHs in the analytical system was calculated by considering a sample volume of 360 L for the smoke samples and converting them into mass concentration. Excluding NAP, the MDLs for all PAHs were below 30 pg m^−3^ regardless of the sorbent tube type, which is well below the annual average air quality target of 1 ng m^−3^ for BaP set by the WHO and EU. The MDLs for PAHs with three or more aromatic rings ranged from 3.12 pg m^−3^ (Chrys, Carbopack C) to 12.5 pg m^−3^ (FLU, quartz wool), demonstrating the high sensitivity and suitability of the system for detecting trace-level PAHs.

### 3.2 Evaluation of PAH mass concentrations in cooking smoke based on different grilling methods (Exp 1)

**Fig. 3:**
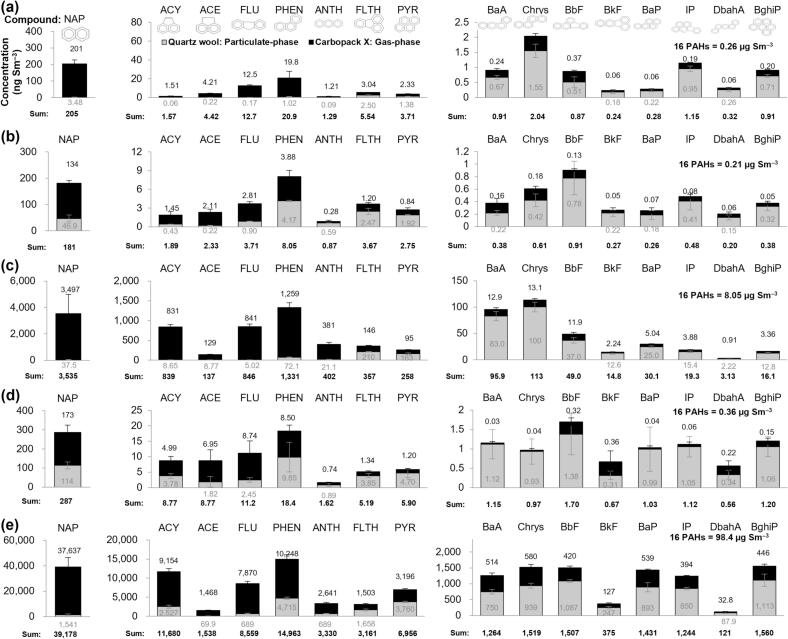
Comparison of PAH mass concentrations in cooking smoke based on different grilling methods (Exp 1). (a) Amb, (b) G, (c) C, (d) G-P-P, and (e) C-G-P.

The mass concentrations of the PAHs emitted during various grilling were quantified and categorized into particulate and gas phases (Fig. 3). The total PAH concentration (Σ16 PAHs) in the ambient air (Amb sample) was 0.26 μg Sm^−3^, with NAP and PHEN identified as the predominant species at 205 ng Sm^−3^ and 20.9 ng Sm^−3^, respectively. The BaP concentration of 0.28 ng Sm^−3^ quantified in the Amb sample was substantially below the WHO and EU air quality guidelines of 1 ng Sm^−3^. The PAH concentration in the gas flame (G) sample was 0.21 μg Sm^−3^, which was approximately 0.05 μg Sm^−3^ lower than that of the Amb sample. This marginal difference was considered statistically insignificant given the analytical variability, indicating that gas combustion alone does not contribute notably to PAH emissions. However, when pork belly was cooked in a frying pan over a gas flame (G-P-P), the total PAH concentration increased to 0.36 μg Sm^−3^, representing a 0.1 μg Sm^−3^ elevation compared to the G and Amb samples. This increase confirmed that the cooking process itself serves as a source of PAHs. The majority of this elevation in the G-P-P sample was attributed to lighter PAHs with four or fewer aromatic rings, including NAP (287 ng Sm^−3^), PHEN (18.4 ng Sm^−3^), FLU (11.2 ng Sm^−3^), ACE (8.77 ng Sm^−3^), ACY (8.76 ng Sm^−3^), and PYR (5.90 ng Sm^−3^). Notably, particulate-phase NAP increased by 67.2 ng Sm^−3^ in the G-P-P sample as compared to the G sample, indicating the enhanced generation of particulate-associated light PAHs during the cooking process.

For the charcoal flame (C) sample, the total PAH concentration reached 8.05 μg Sm^−3^, which was 30-fold and 22-fold higher than the levels observed in the Amb and G-P-P samples, respectively. NAP remained the most abundant PAH in sample C (3535 ng Sm^−3^), followed by PHEN (1331 ng Sm^−3^), FLU (846 ng Sm^−3^), and ACY (839 ng Sm^−3^). The BaP concentration in sample C reached 30.1 ng Sm^−3^, marking a significant elevation. Furthermore, the combined concentration of high-molecular-weight PAHs with five or more aromatic rings in sample C (132 ng Sm^−3^) was more than 21 times higher than that observed in the G-P-P sample (6.28 ng Sm^−3^). These results demonstrate that, while grilling increases the concentrations of lighter PAHs, the type of fuel exerts a significant effect on total emission levels, particularly for high-molecular-weight species such as BaP.

Grilling pork belly over charcoal (C-G-P) resulted in a dramatic surge in PAH emissions, with concentrations reaching an extraordinary 98.4 μg Sm^−3^—over 12 times higher than those of the charcoal flame alone (sample C). NAP remained the dominant species at 35,643 ng Sm^−3^, followed by PHEN (13,632 ng Sm^−3^), ACY (10,841 ng Sm^−3^), and FLU (7713 ng Sm^−3^). Notably, high-molecular-weight PAHs with six aromatic rings, such as IP and BghiP, exhibited the most pronounced increases of 6360 and 9570%, respectively, compared to sample C. BaP also showed a dramatic rise of 6360%, reaching 1431 ng Sm^−3^ in the C-G-P sample, which is more than 1400 times the permissible limit specified in the WHO and EU guidelines.

From a toxicological perspective, BaP is the most critical compound among the 16 PAHs due to its classification as a Group 1 human carcinogen. Although NAP was the most abundant compound by mass in the C-G-P sample, its toxic potency is several orders of magnitude lower than that of BaP. The disproportionate increase in BaP levels during charcoal grilling as compared to gas-based methods indicates that the inhalation risk in such environments is primarily dictated by BaP. Furthermore, other high-molecular-weight species, such as DbahA, which possess high toxic equivalency factors, contributed to the overall increase in smoke toxicity. The synergy between the lipid pyrolysis of meat fat and the incomplete combustion of the charcoal fuel emerged as the primary driver for the substantial emissions of BaP and other high-molecular-weight PAH observed during charcoal grilling.

### 3.3 Evaluation of PAH mass concentrations in charcoal smoke based on different charcoal types (Exp 2)

**Fig. 4:**
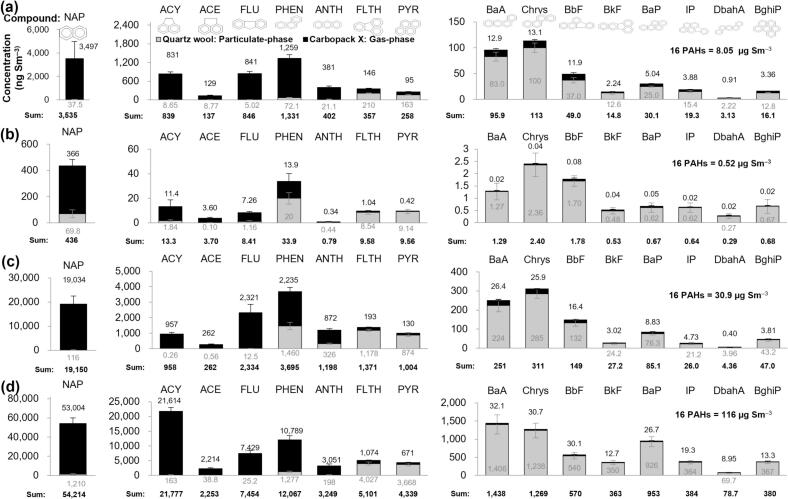
Comparison of PAH mass concentrations in charcoal smoke based on different types of charcoal (Exp 2). (a) C, (b) HC, (c) PC, and (d) CC.

**Fig. 5:**
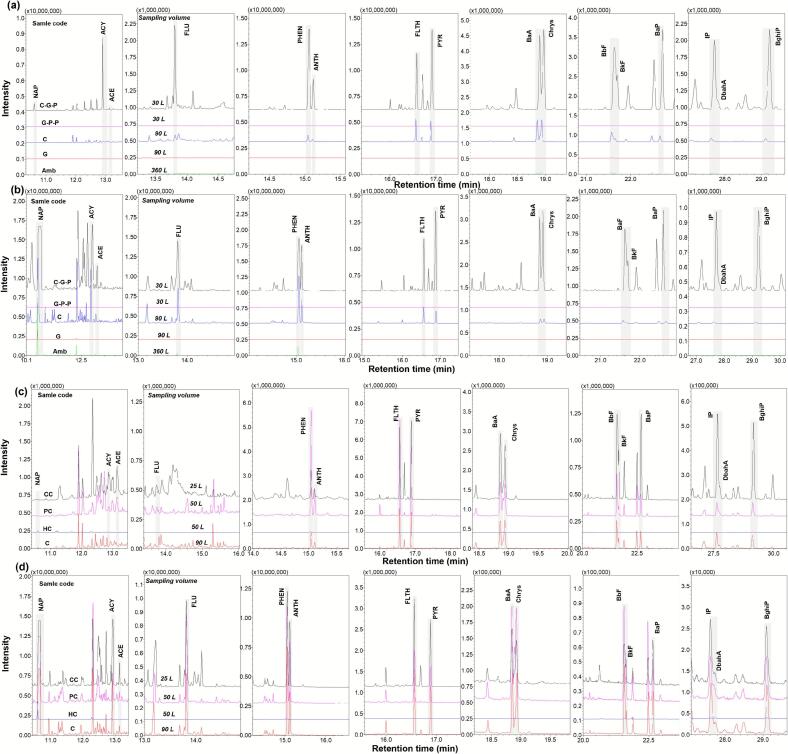
Chromatograms showing PAH analysis in smoke and air samples. (a) Exp 1-Quartz wool, (b) Exp 1-Carbopack C, (c) Exp 2-Quartz wool, and (d) Exp 2-Carbopack C.

As Exp 1 demonstrated that charcoal combustion itself is a prolific source of PAHs, Exp 2 was conducted to evaluate how varying charcoal types influence the magnitude of these emissions (Fig. 4). Sample C utilized here was identical to that in Exp 1. The comparative chromatograms for all samples are presented in Fig. 5. Initial results showed that the total PAH concentration (*n* = 16) for sample C (hardwood, Gangwon-do) was 8.05 μg Sm^−3^, whereas the HC sample (hardwood, Chungcheongbuk-do) exhibited a significantly lower concentration of 0.52 μg Sm^−3^. Although the HC concentration was approximately double the level observed in the ambient air (Amb), it was markedly lower than that of sample C. All 16 PAHs were found at reduced levels in the HC sample, with the BaP concentration reaching only 0.67 ng Sm^−3^, which remains safely below the WHO and EU annual guidelines.

The more than tenfold difference in PAH emissions between the two hardwood charcoals, despite being produced from the same raw material (oriental oak), underscores the decisive roles of regional wood properties and carbonization conditions. Charcoal production involves thermal decomposition, where parameters such as the peak carbonization temperature and residence time dictate the residual volatile organic content (Antal & Grønli, 2003; Zhao et al., 2018). Higher carbonization temperatures typically facilitate a more thorough removal of volatile matter, resulting in minimal PAH emissions during subsequent combustion. This scenario likely explains the lower concentrations in the HC sample. Furthermore, the density, moisture content, and chemical composition of the wood can vary significantly even within the same species, depending on local climatic conditions, ultimately affecting the final charcoal quality (Antal & Grønli, 2003). A detailed examination of individual PAH profiles, used as a proxy for the total volatile composition, reveals that the C sample consistently retained higher levels of all 16 species, particularly light PAHs such as NAP and PHEN. This confirms that the charcoal produced in Gangwon-do (C) possessed a significantly higher residual volatile load than the HC sample, leading to the observed disparity in combustion emissions.

In contrast to the hardwood samples, the PC (palm) and CC (chaff) charcoals exhibited substantially higher emission profiles. The PC sample yielded a total PAH concentration of 30.9 μg Sm^−3^, representing more than a threefold increase compared to sample C, with NAP dominating the emissions at 19,150 ng Sm^−3^. The CC sample produced the highest total PAH levels in this study at 116 μg Sm^−3^, exceeding even the pork belly grilling smoke (C-G-P) from Exp 1. NAP concentrations in the CC sample reached an extraordinary 54,214 ng Sm^−3^, while the particulate-phase BaP concentration (926 ng Sm^−3^) surpassed that of the C-G-P sample (893 ng Sm^−3^). Mechanistically, PC is often produced from agricultural by-products rich in resins and oils, which act as precursors that significantly enhance PAH formation during burning (Kalasee & Dangwilailux, 2021). Similarly, CC, derived from rice husk chaff, contains substantial inorganic components like silica; these can impede complete combustion, thereby generating vast quantities of PAHs (Zhu et al., 2024).

A separate toxicological analysis across these fuel types further clarifies that the health risk is not strictly proportional to the total PAH mass. For instance, while the PC sample had a lower total PAH mass than the CC sample, its BaP levels remained significantly elevated. Among the 16 targets, BaP and DbahA represent the most critical inhalation hazards due to their superior carcinogenic potency. The fact that the CC sample emitted BaP at levels nearly 1000 times higher than the annual ambient air standard emphasizes its extreme toxicity. This implies that charcoal selection must be guided by the potential to release these highly toxic, high-molecular-weight species rather than total mass alone.

Although this study did not directly grill meat using the HC sample, the correlation between charcoal volatiles (C) and the resulting smoke profile (C-G-P) allows for a predictive assessment of flavor and safety. The characteristic smoky flavor of grilled meat is derived from the thermal degradation of charcoal lignocellulose, which releases volatile aroma compounds such as phenols and guaiacols (Guillen & Ibargoitia, 1998; Jiang et al., 2010). As the C sample possessed a higher residual volatile load, it would likely impart a more intense smoky aroma to the meat compared to the HC sample. However, our results indicate that this sensory preference is inherently linked to elevated inhalation risks. Using a more thoroughly carbonized fuel like HC provides a substantially safer grilling environment at the cost of a milder flavor profile. Consequently, the volatile composition of charcoal acts as a double-edged sword; while it enhances culinary “smokiness”, it simultaneously dictates the magnitude of carcinogenic PAH generation during grilling. Thus, optimizing the carbonization degree is essential to balance sensory quality with toxicological safety.

## 4 Conclusions

This study comprehensively investigated the emission characteristics of PAHs generated from charcoal combustion and the grilling of pork belly. Accurate quantification of trace-level PAHs was achieved using an optimized analytical system based on sorbent tube sampling and TD-GC-MS, which provided high sensitivity for short-term grilling events.

The results revealed that while gas-based cooking produced PAH concentrations comparable to ambient levels (0.26 μg Sm^−3^), charcoal combustion and grilling resulted in disproportionately higher emissions. Specifically, charcoal combustion alone yielded total PAH concentrations of 8.05 μg Sm^−3^, and this was further exacerbated when combined with meat grilling, reaching an extraordinary 98.4 μg Sm^−3^. This dramatic surge is attributed to the synergistic interaction between the lipid pyrolysis of meat fat and the incomplete combustion of charcoal fuel. A pivotal finding of this research is that the toxicological risk of grilling smoke is predominantly dictated by BaP and other high-molecular-weight PAHs, rather than the total mass of the 16 targets. In the charcoal grilling smoke, BaP levels reached 1431 ng Sm^−3^, exceeding the WHO and EU annual ambient air quality guidelines by more than 1400-fold. Furthermore, this study identified the carbonization degree of charcoal as a critical factor influencing these emissions. Hardwood charcoal with a higher residual volatile load and chaff-based charcoal were found to emit significantly higher levels of carcinogenic species, with the chaff-based charcoal alone producing BaP at 953 ng Sm^−3^ due to impeded combustion by its inorganic components.

Finally, this research elucidates an inherent trade-off between culinary quality and toxicological safety. While charcoals with higher volatile content may impart a more intense smoky flavor to the meat, they simultaneously increase the generation of hazardous PAHs. These findings underscore the urgent need for standardized guidelines on charcoal quality and carbonization degree for safer grilling practices. This study provides a scientific basis for mitigating inhalation exposure risks and developing effective emission control strategies in the cooking environment.

## CRediT authorship contribution statement

**Yong-Hyun Kim:** Writing – review & editing, Writing – original draft, Visualization, Supervision, Resources, Methodology, Formal analysis, Conceptualization. **Sung-Hwan Kim:** Visualization, Validation, Methodology.

## Declaration of competing interest

The authors declare that they have no known competing financial interests or personal relationships that could have appeared to influence the work reported in this paper.

## Data Availability

Data will be made available on request.

## Appendix A Supplementary data

Supplementary material Image 1
