# Supplementary material for: Evaluation of polycyclic aromatic hydrocarbon (PAH) mass concentrations in smoke generated during pork belly grilling over charcoal
Source: Food Chem X. 2026 Jan 19;34:103558. doi: 10.1016/j.fochx.2026.103558 (PMC12857362; doi:10.1016/j.fochx.2026.103558)
Supplement: Supplementary file 1 — Supplementary material [file mmc1.pdf]

Supplementary Material

Evaluation of polycyclic aromatic hydrocarbon (PAH) mass concentrations in smoke generated during pork belly grilling over charcoal

Yong-Hyun Kim[a,b,c,1,*], Sung-Hwan Kim[d,1]

[a]*Department of Environment & Energy, Jeonbuk National University, 567 Baekje-daero, Deokjin-gu, Jeonju-si, Jeonbuk State 54896, Republic of Korea*

[b]*Department of Environmental Engineering, Jeonbuk National University, 567 Baekje-daero, Deokjin-gu, Jeonju-si, Jeonbuk State 54896, Republic of Korea*

[c]*Soil Environment Research Center, Jeonbuk National University, 567 Baekje-daero, Deokjin-gu, Jeonju-si, Jeonbuk State 54896, Republic of Korea*

[d]*Jeonbuk Branch Institute, Korea Institute of Toxicology, Jeongeup-si, Jeonbuk State 56212, Republic of Korea*

* ***Corresponding author***: *Yong-Hyun Kim, Ph.D.; e-mail: ykim84@jbnu.ac.kr*

[1]These authors contributed equally to this work.

Table S1. List of 16 PAHs selected for this study.

Order	Compounds		Number of aromatic rings	MW (g mol^{-1})	Density (g cm^{-3})	Boiling point (°C)	Chemical formula	CAS number	Mass spectra[a] (m/z)
	Full name	Abbreviation							
1	Naphthalene	NAP	2	128	1.14	218	$C_{10}H_{18}$	91-20-3	128
2	Acenaphthylene	ACY	2	152	0.90	280	$C_{12}H_{8}$	208-96-8	152
3	Acenaphthene	ACE	2	154	1.22	279	$C_{12}H_{10}$	83-32-9	154
4	Fluorene	FLU	3	166	1.20	295	$C_{13}H_{10}$	86-73-7	166
5	Phenanthrene	PHEN	3	178	1.18	332	$C_{14}H_{10}$	85-01-8	178
6	Anthracene	ANTH	3	178	1.25	340	$C_{14}H_{10}$	120-12-7	178
7	Fluoranthene	FLTH	4	202	1.25	375	$C_{16}H_{10}$	206-44-0	202
8	Pyrene	PYR	4	202	1.27	404	$C_{16}H_{10}$	129-00-0	202
9	Benzo[a]anthracene	BaA	4	228	1.19	340	$C_{18}H_{12}$	56-55-3	228
10	Chrysene	Chrys	4	228	1.27	448	$C_{18}H_{12}$	218-01-9	228
11	Benzo[b]fluoranthene	BbF	5	252	1.29	481	$C_{20}H_{12}$	205-99-2	252
12	Benzo[k]fluoranthene	BkF	5	252	1.29	480	$C_{20}H_{12}$	207-08-9	252
13	Benzo[a]pyrene	BaP	5	252	1.24	495	$C_{20}H_{12}$	50-32-8	252
14	Indeno[1,2,3-cd]pyrene	IP	6	276	1.38	536	$C_{22}H_{12}$	193-39-5	276
15	Dibenzo[a,h]anthracene	DbahA	5	278	1.23	524	$C_{22}H_{14}$	53-70-3	278
16	Benzo[g,h,i]perylene	BghiP	6	276	1.38	500	$C_{22}H_{12}$	191-24-2	276

[a]Mass spectra selected for the EIC-base analysis

Table S2. Operational conditions for analysis of 16 selected PAHs.

Order	Parameter		Setting value
A. Thermal desorber (TD-20, Shimadzu, Japan)			
1	Sampling tube (for desorption of PAHs)	Flow rate	100 mL min^{-1} (to cold-trap)
2		Desorption Temp.	290 °C (for 7 min)
3	Cold-trap (for adsorption and desorption of PAHs)	Trap tube	Silicosteel
4		Trap size	Length: 100 mm, OD: 3.2 mm, and ID: 2 mm
5		Adsorbent	Quartz wool 10 mg + Tenax TA 50 mg
6		Adsorption temp.	5 °C
7		Desorption flow rate	16 mL min^{-1} (to GC)
8		Desorption temp.	300 °C (for 5 min)
9	Carrier gas system	Carrier gas type	Helium (purity > 99.9999%)
11		Carrier gas flow rate	2 mL min^{-1} (constant)
12		Split flow rate	10 mL min^{-1}
13		Purge gas flow rate	2 mL min^{-1}
B. Gas chromatography (GC-2010, Shimadzu, Japan)			
14	Column	Column type	DB-5MS (Agilent J&W, USA)
15		Column size	Length: 30 m, ID: 0.25 mm, and film thickness: 0.25 μm
16	Oven	Initial temp.	80 °C (for 5 min)
17		Ramping temp.	20 °C min^{-1}
18		Final temp.	300 °C (for 24 min)
19		Total program time	40 min
C. Mass spectrometry (GCMS-QP2010 ultra, Shimadzu, Japan)			
20	Ionization	Ionization mode	Electron ionization (EI, 70ev)
21		Ion source temp.	280 °C
22		Interface temp.	280 °C
23	Detection	Total ion chromatogram scan range	35 to 600 m/z
24		Scan speed	1250

Table S3. Working standard preparation for the 16 investigated PAHs

Order	Compound	PS[a] (ng µL^{-1})		Working standard (ng µL^{-1})						
				1st	2nd	3rd	4th	5th	6th	7th
1	NAP	9.930		0.099	0.199	0.497	0.993	1.986	4.965	9.930
2	ACY	9.920		0.099	0.198	0.496	0.992	1.984	4.960	9.920
3	ACE	9.930		0.099	0.199	0.497	0.993	1.986	4.965	9.930
4	FLU	9.820		0.098	0.196	0.491	0.982	1.964	4.910	9.820
5	PHEN	10.178		0.102	0.204	0.509	1.018	2.036	5.089	10.178
6	ANTH	9.900		0.099	0.198	0.495	0.990	1.980	4.950	9.900
7	FLTH	9.950		0.100	0.199	0.498	0.995	1.990	4.975	9.950
8	PYR	9.930		0.099	0.199	0.497	0.993	1.986	4.965	9.930
9	BaA	9.850		0.099	0.197	0.493	0.985	1.970	4.925	9.850
10	Chrys	9.984		0.100	0.200	0.499	0.998	1.997	4.992	9.984
11	BbF	9.973		0.100	0.199	0.499	0.997	1.995	4.987	9.973
12	BkF	9.950		0.100	0.199	0.498	0.995	1.990	4.975	9.950
13	BaP	9.975		0.100	0.200	0.499	0.998	1.995	4.988	9.975
14	IP	9.970		0.100	0.199	0.499	0.997	1.994	4.985	9.970
15	DbahA	9.900		0.099	0.198	0.495	0.990	1.980	4.950	9.900
16	BghiP	9.940		0.099	0.199	0.497	0.994	1.988	4.970	9.940
Mixing recipe: in volume (µL)			PS	20	40	100	200	400	1,000	2,000
			Methanol	1,980	1,960	1,900	1,800	1,600	1,000	0
			Total	2,000	2,000	2,000	2,000	2,000	2,000	2,000

[a]Primary standard (PS): PAH Calibration Mix (Supelco, USA).
